# Acoustic Radiation Force Impulse Imaging for the Differentiation of Benign and Malignant Lymph Nodes: A Systematic Review and Meta-Analysis

**DOI:** 10.1371/journal.pone.0166716

**Published:** 2016-11-17

**Authors:** Peige Zhang, Li Zhang, Shaoping Zheng, Cheng Yu, Mingxing Xie, Qing Lv

**Affiliations:** Department of Ultrasound, Union Hospital, Tongji Medical College, Huazhong University of Science and Technology, Wuhan, 430022, China; University of Cincinnati College of Medicine, UNITED STATES

## Abstract

**Objective:**

To evaluate the overall performance of acoustic radiation force impulse imaging (ARFI) in differentiating between benign and malignant lymph nodes (LNs) by conducting a meta-analysis.

**Methods:**

PubMed, Embase, Web of Science, the Cochrane Library and the China National Knowledge Infrastructure were comprehensively searched for potential studies through August 13th, 2016. Studies that investigated the diagnostic power of ARFI for the differential diagnosis of benign and malignant LNs by using virtual touch tissue quantification (VTQ) or virtual touch tissue imaging quantification (VTIQ) were collected. The included articles were published in English or Chinese. Quality Assessment of Diagnostic Accuracy Studies-2 (QUADAS-2) was used to evaluate the methodological quality. The pooled sensitivity, specificity, and the area under the summary receiver operating characteristic (SROC) curve (AUC) were calculated by means of a bivariate mixed-effects regression model. Meta-regression analysis was performed to identify the potential sources of between study heterogeneity. Fagan plot analysis was used to explore the clinical utilities. Publication bias was assessed using Deek’s funnel plot.

**Results:**

Nine studies involving 1084 LNs from 929 patients were identified to analyze in the meta-analysis. The summary sensitivity and specificity of ARFI in detecting malignant LNs were 0.87 (95% confidence interval [CI], 0.83–0.91) and 0.88 (95% CI, 0.82–0.92), respectively. The AUC was 0.93 (95% CI, 0.90–0.95). The pooled DOR was 49.59 (95% CI, 26.11–94.15). Deek’s funnel plot revealed no significant publication bias.

**Conclusion:**

ARFI is a promising tool for the differentiation of benign and malignant LNs with high sensitivity and specificity.

## Introduction

The involment of lymph nodes (LNs) has been demonstrated to be an independent risk factor for local tumor recurrence [1, 2] as well as the most undesirable prognostic factor [3]. Therefore, evaluation of the LNs status is of crucial significance for predicting the prognosis and determining proper treatment protocols in clinical practice [4]. Ultrasonography, compared with computed tomography and magnetic resonance imaging, has proved to be a valuable and cost-effective imaging technique for the differentiation of LNs [5]. However, it is still difficult for the differential diagnosis even combined with color Doppler imaging [6].

Acoustic Radiation Force Impulse (ARFI) imaging is a novel elastography modality which is integrated into a conventional ultrasound machine and could assess the stiffness of tissues quantitatively without external compression in an operator-dependent manner [7]. It could generate focused high-intensity, short-duration acoustic radiation forces by a ultrasound transducer and track the wave propagation as well as the localized displacements in a region of interest (ROI) to compute the value of shear wave velocity (SWV) expressed in the unit of m/s. A higher value of SWV means the tissue is more stiffer. Virtual touch tissue quantification (VTQ) and virtual touch tissue imaging quantification (VTIQ) represent two types of ARFI-generated quantitative techniques. VTQ can calculate the SWV of the tissue from 0 to 8.4 m/s by scaling the time to peak displacement at every lateral location. As a two-dimensional shear wave imaging technique, VTIQ could display color-coded images and detect pulse sequences that can measure SWV from 0.5 to 10 m/s in multiple locations with multiple ROIs placed on the elastogram [8]. Besides the SWV, VTIQ is capable of obtaining quality, travel time and displacement [9].

Several meta-analyses [10–14] of differentiating between benign and malignant LNs have been published in recent years, however, as far as we know, there still lacks a systematic evaluation of ARFI with VTQ and VTIQ for the differential diagnosis of LNs. As a consequence, we conducted this systematic review and meta-analysis to investigate the performance of ARFI using VTQ or VTIQ in the diagnosis of LNs.

## Material and Methods

The meta-analysis was carried out according to the Preferred Reporting Items for Systematic Reviews and Meta-Analyses (PRISMA) checklist (S1 PRISMA Checklist.).

### Search strategy

We searched systematically for the potential literatures up to August13^th^, 2016 in several electronic databases: Pubmed, Embase, Web of Science, the Cochrane Library and the China National Knowledge Infrastructure (CNKI) using the following terms: ("shear wave elastography" OR "acoustic radiation force impulse" OR ARFI OR "virtual touch tissue quantification" OR "virtual touch tissue imaging quantification") AND "lymph nodes". The language was restricted to English or Chinese. What’s more, we also scrutinized the bibliographies of the relevant studies manually so as to identify more potential articles.

### Study selection

Studies which met the following inclusion criteria were considered to be eligible for the meta analysis.

1. evaluated the value of ARFI for identifying LNs by VTQ or VTIQ.
2. used the appropriate reference standard: histopathologic examination (surgery, core biopsy) or cytological examination (Fine-Needle Aspiration).
3. provided enough data to construct 2×2 contingency tables (containing true-positive, false-positive, true-negative and false-negative diagnostic results).

Publications that did not offer the original data such as case reports, editorials, letters, reviews and meta-analysis were excluded. Duplications or updated literatures were excluded. If two or more studies were performed in the same medical institution by the same author, the study with smaller sample size was excluded.

The eligibility of the articles was estimated independently by two authors (PGZ and LZ) with the criteria mentioned above. Discrepancies between the two authors were resolved by consensus or judged by a third author (SPZ).

### Data extraction and quality assessment

The following information were extracted from the eligible studies: first author’s name, publication year, country, number of the patients available for analysis, mean age of the participants, location of the LNs, number of malignant/benign LNs, reference standard, the diagnostic results by VTQ or VTIQ: cut-off values, sensitivity, specificity. The Quality Assessment of Diagnostic Accuracy Studies-2 (QUADAS-2) tool was applied to assess the methodological quality of the selected studies in this meta-analysis. The QUADAS-2 tool was performed in Review Manager 5.2.

### Statistical analysis

To determine the heterogeneity among all the studies, Cochrane Q statistics and inconsistency index (I^2^) were used. Cochrane Q Statistics P value <0.1 suggested the existence of heterogeneity [15]. As for the I^2^ index, we adopted the interpretation of the Cochrane Handbook for Systematic Reviews of Interventions Version 5.1.0: the I^2^ values of 0% to 40%, 30% to 60%, 50% to 90%, 75% to 100% indicated the heterogeneity might be not important, moderate, substantial, considerable, respectively [16]. Furthermore, meta-regression was performed to explore the sources of heterogeneity according to the following predefined covariates: language (English versus Chinese), sample size (<110 LNs versus >110 LNs), prevalence of malignant LNs (<50% versus >50%), cut-off value (<2.6 m/s versus >2.6 m/s), location of LNs (cervical versus others), index test (VTQ versus VTIQ). A p-value <0.05 indicated significance. Threshold effect was analyzed with SROC space and Spearman correlation coefficient [17]. The representation of a typical "shoulder arm" pattern in a SROC space and a strong positive correlation between the log of sensitivity and log of 1-specificity may represent the presence of threshold effect [18, 19]. Moreover, a considerable threshold effect existed if the Spearman correlation coefficient >0.6. The diagnostic accuracy of ARFI in differentiation of malignant and benign LNs was estimated by pooled sensitivity, specificity and the diagnostic odds ratio (DOR) with corresponding 95% confidence interval (CI) and the summary receiver operating characteristic (SROC) curve. The area under the curve (AUC) was identified as a global measure of test performance. The following guidelines have been suggested for interpretation of AUC values: low (0.5> = AUC < = 0.7), moderate (0.7> = AUC < = 0.9), or high (0.9 > = AUC < = 1) accuracy [20]. A bivariate mixed-effects regression model was carried out to synthesize data. Besides, Deek’s funnel plot was utilized to examine the publication bias of the selected studies and a P-value < 0.10 indicated significant asymmetry [21].

In addition, the clinical utility of diagnostic test can be evaluated using the likelihood ratios (LR) to calculate post-test probability based on Bayes’ theorem [22]. Fagan plot which showed the relationship between the pre-test probability, the LR, and the post-test probability was used to evaluate pre-test probabilities of 25%, 50%, 75% versus corresponding post-test probabilities following a "positive" or "negative" ARFI results based on the overall sensitivity and specificity [23, 24]. "Positive" or "negative" ARFI results were defined as all results above or below the optimal cut-off value for malignant LNs, shown in each individual study [11].

The Midas module of Stata 14.0 and Meta-disc 1.4 was used to perform all the statistical analyses.

## Results

### Literature search

**Fig 1:**
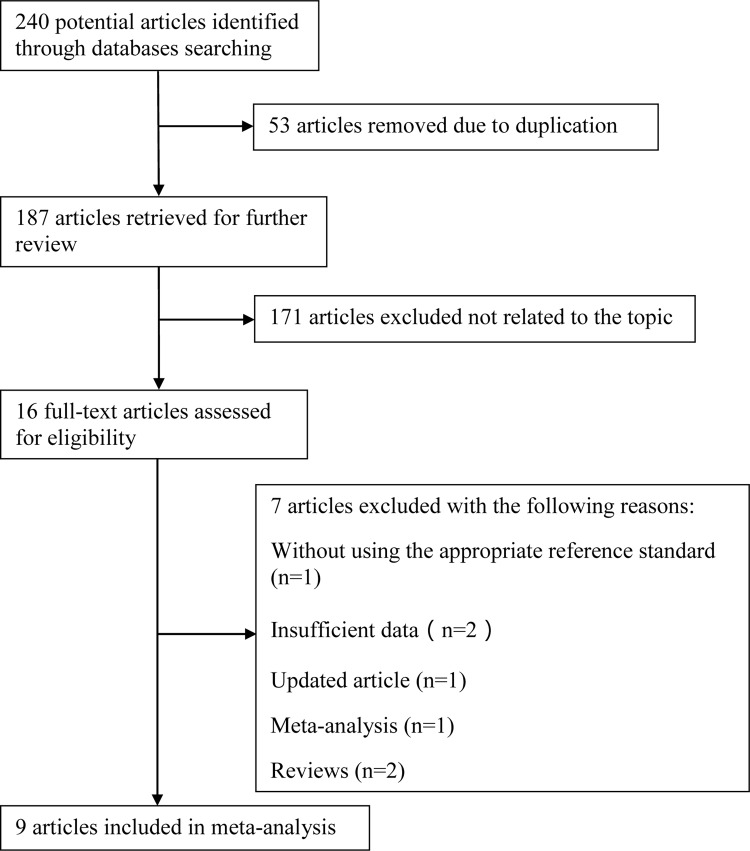
A flow diagram of screening eligible studies. The flow chart illustrated the process of reviewing studies and the numbers of studies identified at each stage. Nine studies were included in this meta-analysis.

A flow chart diagraming the process of study selection is illustrated in Fig 1. The initial literature search yielded 240 manuscripts with the predefined search terms. After removing duplicates, 187 articles were retrieved, of which 171 studies were then excluded by screening the titles and abstracts as not related to the topic. We assessed the eligibility of the remaining 16 articles: one article was excluded because of using the inappropriate reference standard, two articles did not report the interesting data, one meta-analysis and two reviews were also eliminated, two articles including a similar set of patients were performed in the same medical center by a same author, and the study with smaller sample size was excluded. Ultimately, nine full-text articles [25–33] fulfilled the inclusion criteria and were considered eligible for the meta-analysis.

### Study characteristics

The main characteristics extracted from the selected studies are listed in Table 1. The studies included in this meta-analysis were published from 2013 to 2016. A total of 929 patients with 1084 LNs (469 malignant, 615 benign) were investigated. Patients’ age ranged from 1–85 years, while we failed to acquire this information from two articles [25, 27]. Three studies [25, 30, 31] did not concern about the gender distribution, and 47.7% were males for the remanent studies. The number of LNs ranged from 42 to 270 and the mean number was 120. Three articles [25, 26, 32] calculated shear wave velocities (m/s) by VTIQ, and VTQ was used for the remaining six articles.

**Table 1:** Main characteristics of the studies included in the meta-analysis.

Author year	Country	No. of patients	Mean age (years)	Location of LNs	Malignant/benign LNs	Reference standard	Cut-off value(m/s)	Se%	Sp%	Index test
Azizi G 2016	USA	231	NA	Cervical	54/216	FNAB cytology or surgical pathology	2.93	92.59	75.46	VTIQ
Cheng KL 2015	South Korea	100	52.7	Cervical	57/43	Cytopathology	3.34	78.9	74.4	VTIQ
Chen SQ 2015	China	113	NA	Cervical,inguinal,axillary	53/60	Histopathology	4.645	92.5	96.7	VTQ
Fujiwara T 2013	Japan	19	63.7	Cervical	20/22	Histopathology	1.9	95	81.8	VTQ
Liu LJ 2015	China	65	47.6	Cervical	21/79	Histopathology	1.9	82.2	90	VTQ
Meng DL 2015	China	78	43.2	Cervical	43/35	Histopathology	3.98	92.9	90.1	VTQ
Meng W 2013	China	123	40.8	Cervical	94/87	Histopathology	2.595	82.9	93.1	VTQ
Zhang JP 2015	China	56	49.6	Cervical	35/21	Histopathology	3.14	77.1	85.7	VTIQ
Zhen X 2015	China	144	47.3	Cervical	92/52	Histopathology	2.507	89.13	90.38	VTQ

LNs: lymph nodes; Se: Sensitivity; Sp: Specificity; FNAB: fine-needle aspiration biopsy; NA: not available; VTQ: virtual touch tissue quantification; VTIQ: virtual touch tissue imaging quantification.

### Methodology quality assessment

**Fig 2:**
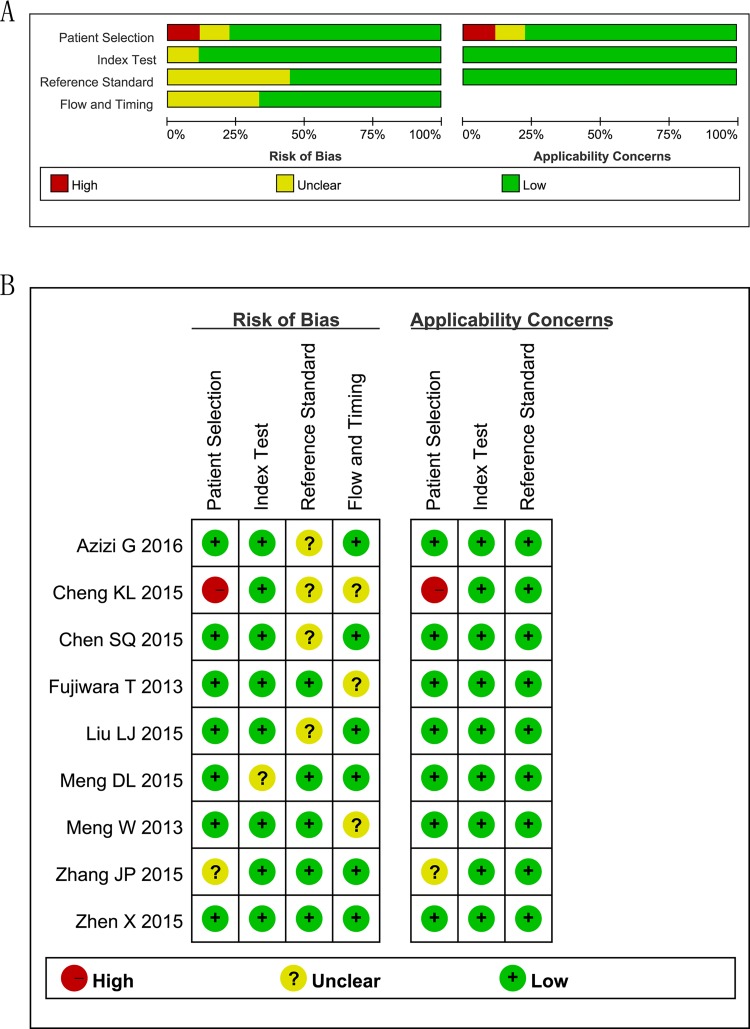
Assessment of Methodological Quality with the Quality Assessment of Diagnostic Accuracy Studies-2 tool. (A) Risk of bias and applicability concerns graph: review author’s judgments about each domain presented as percentages across the selected studies. (B) Risk of bias and applicability concerns summary: review authors’ judgments about each domain for each included study.

The quality assessment of the included studies by using QUADAS-2 tool is shown in Fig 2. Roughly speaking, the quality was satisfactory.

### Diagnostic performance of ARFI

**Fig 3:**
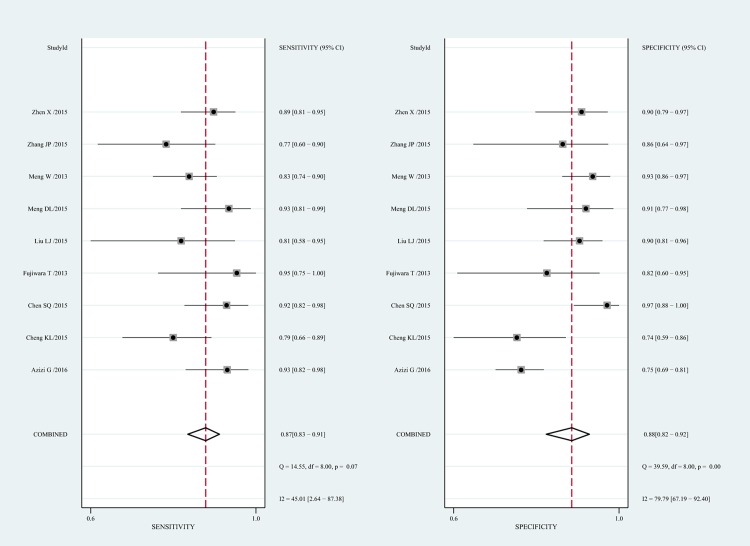
Forest plots for sensitivity and specificity of Acoustic Radiation Force Impulse (ARFI) imaging for the differentiation of benign and malignant LNs. Horizontal lines represent 95% CIs of the individual studies.

**Fig 4:**
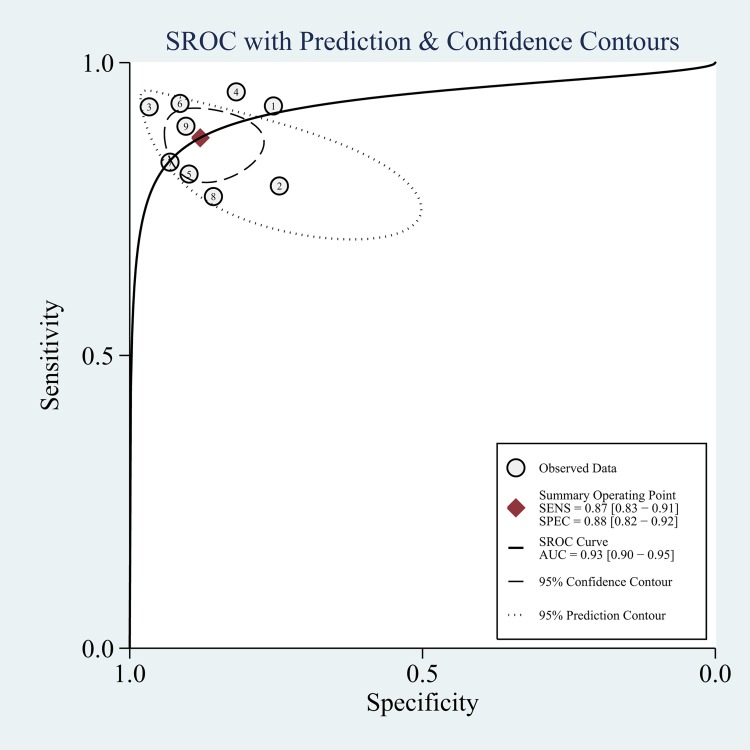
The Summary Receiver Operating Characteristic (SROC) curve for ARFI in differential diagnosis for benign and malignant LNs. SENS: sensitivity; SPEC: specificity; SROC: summary receiver operating characteristic curve; AUC: area under the SROC curve.

The diagnostic performance of ARFI in distinguishing malignant LNs was evaluated by pooling the sensitivity, specificity and the DOR with a bivariate mixed-effects regression model. Sensitivity and specificity represent the rates of a test correctly identifying patients with and without disease [34]. Fig 3 displayed the forest plots of the pooled indices. The pooled sensitivity and specificity were 87% (95% CI, 83–91%) and 88% (95%CI, 82–92%), respectively. As shown in Fig 4, a symmetrical SROC curve was depicted, and the AUC was 0.93 (95% CI, 0.90–0.95) which indicated a high diagnostic accuracy. The pooled DOR describes the odds of positive test results in participants with disease compared with the odds of positive test results in those without disease, and its value remains relatively constant compared to pooled sensitivity and specificity [35]. The DOR of ARFI in detecting LNs was found to be 49.59 (95% CI, 26.11–94.15; S1 Fig).

### Heterogeneity tests

Moderate heterogeneity was observed in pooled sensitivity (Cochrane Q test = 14.55, df = 8, p = 0.07, I^2^ = 45.01), whereas substantial heterogeneity was found in pooled specificity (Cochrane Q test = 39.59, df = 8, p = 0.00, I^2^ = 79.79) (Fig 3). There was no significant threshold effect in view of the Spearman correlation coefficient (r = -0.15, p-value = 0.70), which suggested that other factors might result in the heterogeneity among the studies instead of threshold effect. According to the meta-regression analysis, the differences in language, sample size, prevalence of malignant LNs, cut-off value, location of LNs, index test had nothing to do with heterogeneity with a p-value of 0.65, 0.70, 0.61, 0.47, 0.70, 0.36, respectively.

### Subgroup analysis between VTQ and VTIQ

There were three studies included in the VTIQ subgroup and six studies in the VTQ subgroup. Pooled estimates for sensitivity, specificity, and the AUC were lower in the VTIQ subgroup than that in the VTQ subgroup. Results of the subgroup analyses are presented in Table 2.

**Table 2:** Subgroup analysis.

Subgroup		Studies, n	LNs, n	Sensitivity(95% CI) I^2^, %	Specificity(95% CI) I^2^, %	AUC
All studies		9	1084	0.87(0.83–0.91) 45.01	0.88(0.82–0.92) 79.79	0.93
ARFI technique	VTQ	6	658	0.88(0.84–0.92) 22.5	0.92(0.88–0.94) 5.9	0.96
	VTIQ	3	426	0.84(0.77–0.89) 64.7	0.76(0.71–0.81) 0	0.87

LNs: lymph nodes; ARFI: acoustic radiation force impulse; VTQ: virtual touch tissue quantification; VTIQ: virtual touch tissue imaging quantification; CI: confidence interval; AUC: area under the summary receiver operating characteristic curve.

### Publication bias

**Fig 5:**
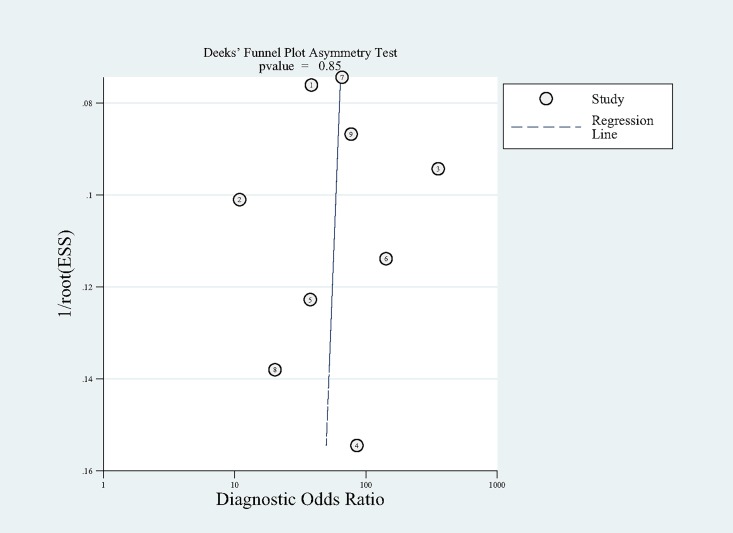
The Deek's Funnel Plot Asymmetry Test for evaluating publication bias among the included studies. No significant publication bias was found in this meta-analysis. Each circle represented an eligible study. ESS = effective sample size.

The Deek’s funnel plot in Fig 5 indicated that no significant publication bias existed among the studies with a p-value of 0.848.

### Fagan plot analysis

**Fig 6:**
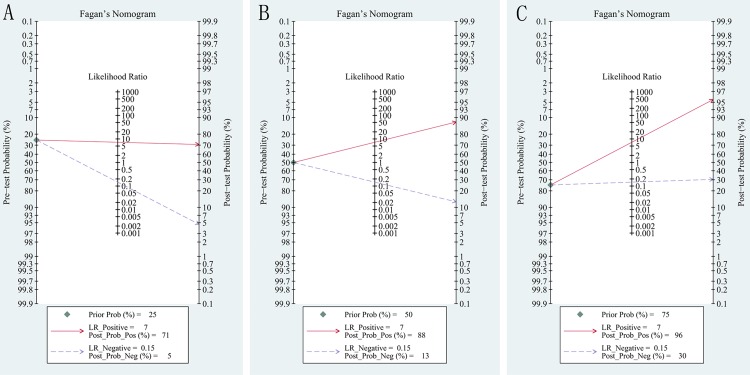
Fagan plot analysis for ARFI in the differentiation of benign and malignant LNs. **(A) pre-test probability at 25%; (B) pre-test probability at 50%; (C) pre-test probability at 75%.** The Fagan plot consisted of a vertical axis on the left with the pre-test probability, an axis in the middle representing the likelihood ratio and a vertical axis on the right representing the post-test probability.

The analysis of Fagan plot testified that ARFI could provide quite informative utility for diagnosing malignant LNs with 88% probability of correct diagnosis following a "positive" measurement and lowering the probability of disease to 13% following a "negative" measurement when the pre-test probability was 50% (Fig 6A). The positive post probabilities were 71% and 96% respectively when the pretest probabilities were 25% and 75%, meanwhile, the negative post probabilities were 5% and 30% (Fig 6B and 6C).

## Discussion

Enlarged LNs could be caused by a variety of diseases such as hyperplasia, infections, lymphoma, granuloma, metastasis, tuberculosis and so on. The status of LNs is the most important prognostic factor for patients with cancer, Thus, it is momentous to identify the nature of LNs from the clinical viewpoint [4, 10].

Fine-needle aspiration cytology (FNAC), as an invasive diagnostic method, is subject to sampling errors and analytic uncertainty despite the efficiency for correct diagnosis [5].

With the emergence of ARFI which is a recently developed modality for assessing tissue stiffness quantificationally, it provides an alternative method to evaluate LNs due to the advantages such as non-invasive, low-cost, accessible, etc.

In this meta-analysis, we investigated the overall performance of ARFI for classifying LNs. Based on the analysis of nine studies containing 1084 LNs from 929 patients, it is persuasive that ARFI had a high accuracy for the identification of benign and malignant LNs with a pooled sensitivity of 0.87 and specificity of 0.88, and the corresponding AUC of 0.93. A recent meta-analysis by Suh CH [13] reported eight studies using shear wave elastography to discriminate 647 cervical LNs from 481 patients. The pooled sensitivity, specificity, and AUC were 0.81, 0.85, 0.88, respectively. However, this meta-analysis combined ARFI and SSI together, and unfortunately, only a total of four articles were focused on the ARFI technique. Our study included a larger number of articles (n = 9) as well as LNs (n = 1084), in addition, our meta-analysis was specially conducted to determine the value of ARFI in differentiating benign and malignant LNs.

Nevertheless, a homogeneity test of sensitivity and specificity showed that Q = 14.55, p = 0.07, I^2^ = 45.01%, and Q = 39.59, p = 0.00, I^2^ = 79.79% which indicated that the heterogeneity among individual studies should not be ignored. There was no evidence that threshold effect existed within the studies (r = -0.15, p-value = 0.70), revealing that there should be other factors that may contribute to the heterogeneity. Disappointingly, meta-regression analyses showed that the differences in language, sample size, prevalence of malignant LNs, cut-off value, location of LNs and index test could not explain the between-study heterogeneity.

Our meta-analysis encompassed two quantitative techniques of ARFI, VTQ and VTIQ. Based on the short-duration acoustic force, local tissue was excited to generate displacement. VTQ is a single-point shear wave velocity (SWV) measurement and can calculate the SWV by scaling the time to peak displacement at each lateral location. VTIQ, as a two-dimensional technique, can display color-coded images and measure the SWV using 256 spatial distributions [8]. In the subgroup analysis, the diagnostic accuracy in the VTQ subgroup was higher than the VTIQ subgroup, and the findings showed that the heterogeneity was not important in the VTQ subgroup with the I^2^ value of 22.5% for the sensitivity and 5.9% for the specificity. However, in the VTIQ subgroup, there was a substantial heterogeneity with the I^2^ value of 64.7% for the sensitivity and for the specificity, the heterogeneity was eliminated. Yang JP, et al [36] published a research comparing the performance of VTIQ with VTQ, and it was found that VTIQ showed better performance for diagnosis of thyroid nodules. Considering that only three articles were included in the VITQ subgroup in our present meta-analysis, more tests focused on the VTIQ technique are necessary in the future.

What’s more, Fagan plot analysis had been used to explore the clinical utilities of ARFI. Our findings showed that ARFI technique could have potential for identifying LNs. When pre-test probability was 50%, the probability of correctly diagnosing LNs was 88% following a "positive" measurement and malignant LNs were found in only 13% of patients following a "negative" measurement.

To the best of our knowledge, our present meta-analysis is the first to evaluate the diagnostic performance of ARFI for detecting malignant LNs specially and systematically. However, our research has several limitations. First, due to the relative few number of studies for the overall performance (n = 9) as well as the subgroup analysis (n = 3 for the VTIQ subgroup, n = 6 for the VTQ subgroup), it may reduce the power to assess the accuracy of ARFI and might result in publication bias and heterogeneity. Second, we did not make a comparison between the ARFI and other imaging tools for distinguishing LNs. Third, in our meta-analysis, Asian studies accounted for the most of the proportion. Therefore, multicenter studies are expected to be conducted from different regions.

## Conclusions

ARFI is useful in differentiating between malignant and benign LNs, and it can be considered as a complement for conventional ultrasonography.

## Supporting Information

S1 PRISMA Checklist (DOC)

S1 Fig Forest plots of diagnostic score and diagnostic odds ratio (DOR) of Acoustic Radiation Force Impulse (ARFI) imaging in identifying benign and malignant LNs. (TIF)
