# Supplementary figures and images for: Acoustic Radiation Force Impulse Imaging for the Differentiation of Benign and Malignant Lymph Nodes: A Systematic Review and Meta-Analysis
Source: PLoS One. 2016 Nov 17;11(11):e0166716. doi: 10.1371/journal.pone.0166716 (PMC5113967; doi:10.1371/journal.pone.0166716)

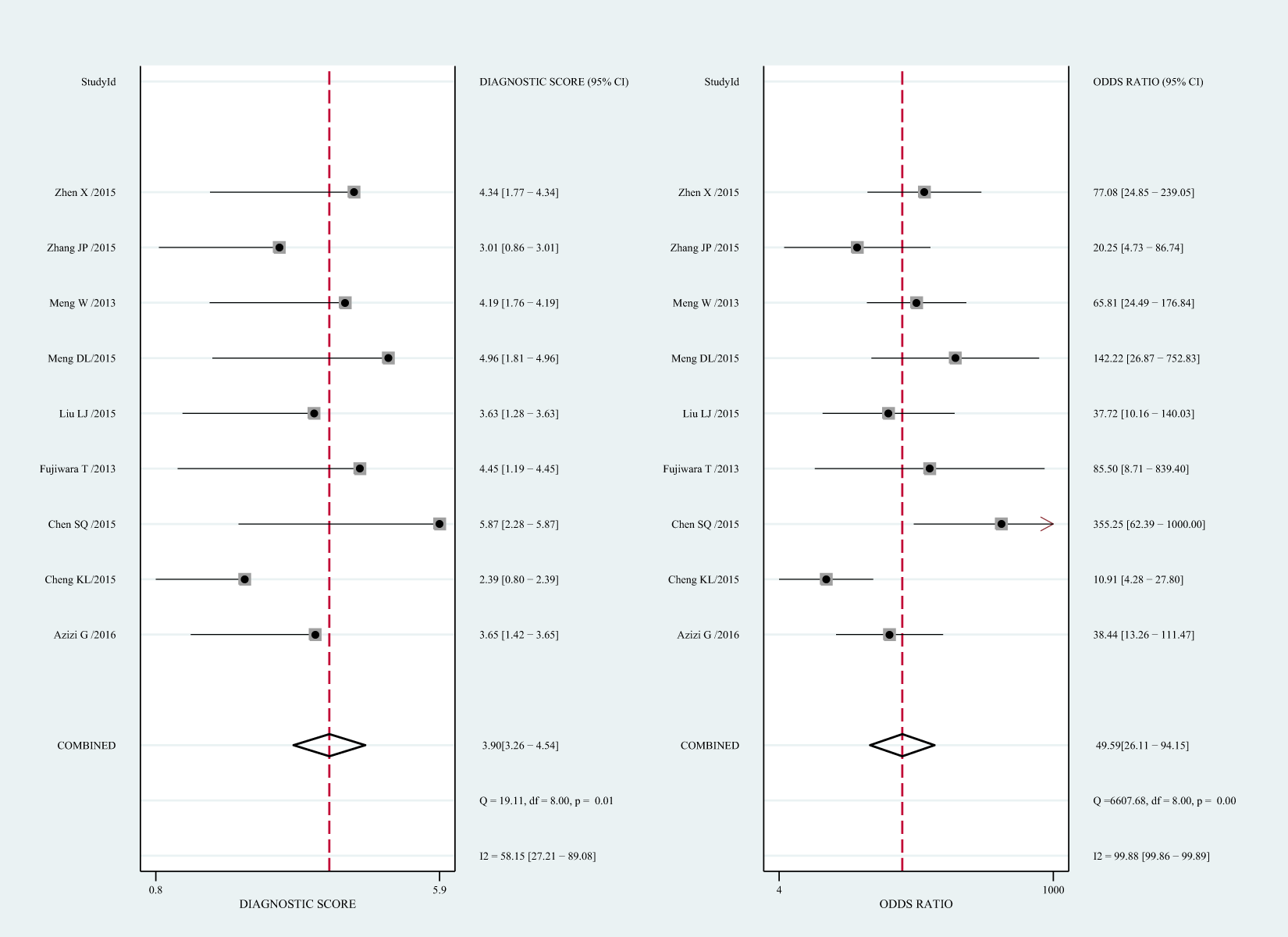

Supplement: S1 Fig — (TIF) [file pone.0166716.s002.tif]
